# Effects of recumbent isometric yoga on the daily functioning level of patients with myalgic encephalomyelitis/chronic fatigue syndrome: a randomized, controlled trial

**DOI:** 10.1186/s13030-025-00339-7

**Published:** 2025-10-10

**Authors:** Takakazu Oka, Battuvshin Lkhagvasuren, Yu Yamada

**Affiliations:** 1 Department of Psychosomatic Medicine, International University of Health and Welfare Hospital, Nasushiobara, Tochigi, Iguchi 537-3 329-2763 Japan; 2 Department of Psychosomatic Medicine, Graduate School of Medical Sciences, Kyushu University, Maidashi 3-1-1, Fukuoka, 812-8582 Japan

**Keywords:** Myalgic encephalomyelitis/chronic fatigue syndrome, Fibromyalgia, Isometric yoga, Fatigue, Treatment

## Abstract

**Background:**

Although seated isometric yoga has been shown to reduce the fatigue and pain of patients with myalgic encephalomyelitis/chronic fatigue syndrome (ME/CFS), some patients who are for the most part bedridden have difficulty practicing it. Many patients with this disease also suffer from fibromyalgia (FM). We developed a recumbent isometric yoga program for patients who were for the most part bedridden, including patients with comorbid FM. The aim of this study was to investigate the effect of this recumbent isometric yoga intervention with such patients.

**Methods:**

This was a randomized, controlled trial of 48 adult patients (7 male, 41 female, age 20–70 years) with ME/CFS without satisfactory improvement after ≥ 3 months of conventional therapy. They were divided randomly into a yoga group (n = 24) and a control group (n = 24). The yoga group received conventional therapy with recumbent isometric yoga practice for ~ 12 weeks (25-min sessions with a yoga instructor at hospital visits and daily in-home sessions). The control group received conventional therapy alone. The effect of recumbent isometric yoga on the level of functioning was assessed by measuring Performance Status (PS). Fatigue was assessed with self-rated questionnaires, including the Chalder Fatigue Scale (FS) and Profile of Mood States (POMS). Adverse events and benefits were recorded for the yoga group.

**Results:**

After the intervention period, the PS score of the yoga group was significantly lower than that of the control group (*P* < 0.001), suggesting an improvement in functioning level. The Chalder FS score decreased in both groups, but the decrease was greater in the yoga group than in the control group (*P* < 0.01). Subgroup analysis showed that the Chalder FS score was reduced significantly only in the yoga group in patients with severe disease (*P* < 0.001) and those with comorbid FM (*P* < 0.01), although the PS scores did not differ significantly. In the yoga group, a single practice session with a yoga instructor significantly reduced fatigue and increased vigor in patients with severe disease and patients with comorbid FM. Patients reported no serious adverse effects and many benefits of recumbent isometric yoga, including improvements in physical symptoms and brain fog, enhanced awareness of their limits to activities that cause post-exertional malaise, and promotion of behavioral changes to live better within their limits.

**Conclusions:**

Recumbent isometric yoga is an effective adjunctive therapy for patients with ME/CFS, including those for the most part bedridden and those who have FM.

**Trial registration:**

University Hospital Medical Information Network (UMIN CTR) UMIN000023472 (Registered Aug. 4, 2016) and UMIN000030051 (Registered Nov. 20, 2017).

## Background

Myalgic encephalomyelitis/chronic fatigue syndrome (ME/CFS) is a debilitating disease characterized by persistent or relapsing unexplained fatigue of at least a six-month duration that is not relieved by rest and causes a substantial reduction in daily activities [1, 2]. It is associated with a variety of symptoms, including post-exertional malaise (PEM), sleep disturbance, pain, neurocognitive impairment (e.g. confusion, impaired concentration, short-term memory consolidation, and difficulty with information processing), and orthostatic intolerance. PEM is an exacerbation of some or all of an individual’s ME/CFS symptoms that occurs after physical or cognitive exertion and leads to a reduction in functional ability [3, 4]. PEM is the hallmark symptom of ME/CFS, and patients often describe it as a “crash”. The health-related quality of life of patients with ME/CFS is severely impaired when compared with healthy controls and other physical or psychiatric conditions [5, 6], with ME/CFS having a significant impact on patients’ physical health and functional levels, for example, in their ability to perform daily activities or work [6].

Currently, yoga is considered a self-help strategy for patients with ME/CFS [2]. For example, the Canadian consensus document on ME/CFS recommends that patients use techniques and practices like yoga to improve balance [2]. Based on our clinical studies described below, the Japanese clinical practice guide for ME/CFS recommended that yoga be considered, but further scientific evidence is needed [7]. When we began our research to investigate the possibility of using yoga to treat patients with ME/CFS, only one case report suggested the usefulness of a yoga-based lifestyle intervention [8]. Therefore, we developed two yoga programs that aimed to improve fatigue without exacerbating symptoms or inducing PEM, and we have been assessing the effects of these programs [9, 10]. The first was a 20-min isometric yoga program with the participant in a seated position (seated isometric yoga). We demonstrated in a randomized, controlled trial (RCT) that practicing seated isometric yoga for two months improved fatigue and pain in patients with ME/CFS who were resistant to conventional therapies [9]. However, patients who had difficulty remaining in a seated position for more than 30 min or who spent most of their time in bed were excluded from this trial because it was difficult for them to practice seated isometric yoga. No yoga program was available for these severe patients. Therefore, we developed a recumbent isometric yoga program so that such patients could practice it in bed. Our pilot study suggested that recumbent isometric yoga is feasible for patients with severe ME/CFS [10]. Furthermore, patients who practiced both seated and recumbent isometric yoga preferred the recumbent yoga program to the seated one because they used less energy and experienced deeper relaxation and comfort [10]. Therefore, we hypothesized that practicing this new isometric yoga program would provide benefits to severely ill patients with ME/CFS. Considering the potential pain-relieving effects, we also hypothesized that the new isometric yoga program would be beneficial for those with comorbid fibromyalgia (FM). That was very interesting to study, because it is reported that 23% [11]—55% [12] of patients with ME/CFS suffer from comorbid FM and that the overall health states of patients with ME/CFS and FM are poorer than those with ME/CFS or FM alone [11, 13]. Furthermore, no non-pharmacological treatment for patients with ME/CFS and comorbid FM has yet been established.

To test these hypotheses, the present RCT was done to investigate the efficacy of a recumbent isometric yoga program in patients with ME/CFS, including severe cases and those with comorbid FM.

## Methods

### Subjects

Participants were outpatients with ME/CFS who visited the Department of Psychosomatic Medicine at the International University of Health and Welfare Hospital or at Kyushu University Hospital without satisfactory improvement, i.e. those who still had difficulties in their daily lives and had asked for further treatment options, after at least three months of conventional therapy administered at these hospitals. Treatments at these hospitals included guidance on self-help strategies so that the patient can engage in appropriate coping; self-monitoring of the disrupted diurnal rhythm of body temperature and sleep–wake cycle followed by their modification; psychotherapy to reduce guilt, self-blame, anxiety, tension or rumination of negative thoughts; and to enhance the awareness of the threshold to exacerbate symptoms or cause PEM, environmental arrangement at home, workplace or school, and symptomatic pharmacotherapy [14–16]. Pharmacotherapy was administered with the aim of improving symptoms such as orthostatic dysregulation/intolerance, including postural orthostatic tachycardia syndrome, sleep disturbance, pain, and those associated with comorbid psychiatric and physical conditions/diseases. In particular, pain was treated as much as possible to allow patients with comorbid FM to lie in bed and practice this isometric yoga program. Participants also satisfied the following inclusion criteria: between 20 and 70 years of age and performance status (PS) between 3 and 8 (Table 1) [17]. Patients who had previously practiced yoga were excluded, as were those whose fatigue was considered due to physical disease such as liver, kidney, heart, respiratory, endocrine, autoimmune, or malignant disease; severe anemia; electrolyte abnormalities; obesity; and/or pregnancy.

**Table 1:** Performance status (PS), i.e. a ME/CFS patient’s level of functioning, was determined by the clinical case definition of ME/CFS in Japan (2017) [17]. The English translation was adopted from Oka and colleagues [18]

0: The subject is able to have a normal social life without fatigue and to function without restrictions.
1: The subject is able to have a normal social life and work, but often feels fatigue.
2: The subject is able to have a normal social life and work, but often needs rest due to general malaise.
3: The subject is unable to engage in social life or work for several days a month because of general malaise and needs to rest at home.
4: The subject is unable to engage in social life or work for several days a week because of general malaise and needs to rest at home.
5: The subject is unable to engage in normal social life or work. The subject is able to do light work, but needs to rest at home several days a week.
6: Light work is possible on good days, but the subject rests at home more than 50% of the week.
7: The subject is able to take care of him/herself and does not need assistance, but normal social activities and light work are not possible.
8: The subject is able to take care of him/herself to a certain extent, but often needs assistance and stays in bed more than 50% of the time during the day.
9: The subject is unable to take care of him/herself at all, requires constant assistance, and stays in bed all day.

^*^In a patient with ME/CFS, the condition must be PS 3 or higher

### Randomization

**Fig. 1:**
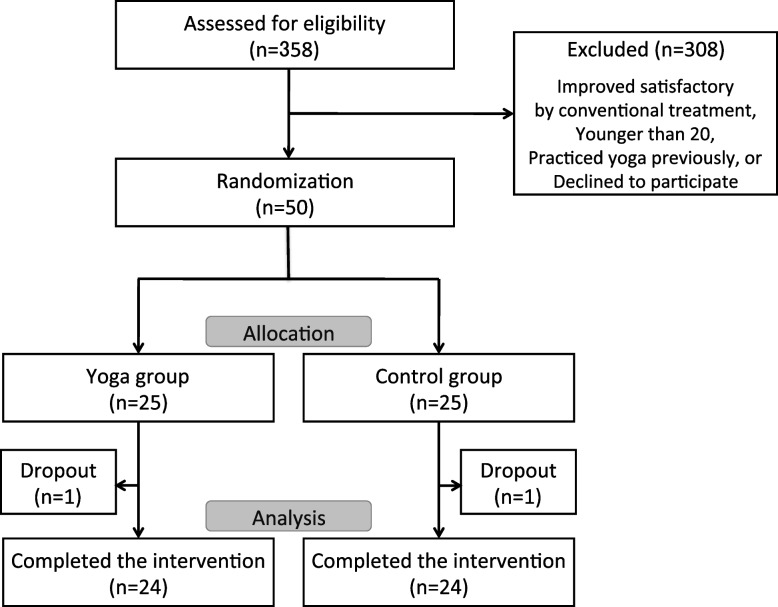
Flow chart outlining participation in this study

Following enrollment, PS and the presence of comorbid FM were evaluated. The patients were then randomized with a computer-generated randomization list to either a twelve-week recumbent isometric yoga program together with conventional therapy group (yoga group, *n* = 25) or a conventional therapy alone group (control group, *n* = 25) (Fig. 1).

#### Diagnosis of ME/CFS and FM

The diagnosis of ME/CFS was made when the following diagnostic criteria was met: the 1994 Fukuda case definition of CFS [1], the 2003 Canadian clinical case definition of ME/CFS [3], the 2011 International Consensus Criteria for ME [19], the 2015 systemic exertion intolerance disease (SEID) [4], and the 2017 clinical case definition of ME/CFS in Japan [17]. The diagnosis of FM was made based on the 2010 diagnostic criteria of the American College of Rheumatology [20].

#### Assessment of PS

The attending physician scored each patient’s PS by rating the disease severity and level of functioning, which ranged from 0 (same level as healthy subjects) to 9 (worst performance status), based on the Japanese clinical diagnostic criteria for ME/CFS (Table 1) [17, 18]. In this study, patients with PS 7 and 8 were classified as severe and those with PS 3, 4, 5, and 6 were classified as mild to moderate.

#### Yoga intervention

Patients in the yoga group practiced recumbent isometric yoga on the day they visited the hospital in a quiet room on a one-to-one basis with an instructor who had more than 30 years of experience as a yoga instructor and more than seven years of experience instructing patients with ME/CFS. The sessions occurred between 2 and 4 pm. Before and after practicing isometric yoga, the supervising doctor checked the patient’s condition and recorded any adverse events or discomfort, such as exacerbation of fatigue, pain, dizziness, or anxiety, that could possibly be caused by practicing isometric yoga. In addition to receiving a private lesson, the participants were asked to practice this program on non-class days if they could, with the aid of a video on YouTube (Isometric yoga program for ME/CFS and long COVID) [21] or a booklet. The overall flow of the yoga program was as shown in the video [21], but the program was modified for each patient based on the opinions of the yoga instructor and the patient's doctor. Most patients visited their doctor every four weeks during the intervention period. During the intervention period, pharmacotherapy was continued, and medication dosages were not changed.

#### Recumbent isometric yoga program

We developed a recumbent isometric yoga program that patients who spend most of their time in bed can practice without exacerbating their symptoms or causing PEM. We also aimed to prevent the occurrence of postural orthostatic tachycardia by avoiding sitting or standing postures. A detailed description of the development of this program has been reported elsewhere [10]. Patients practiced the recumbent isometric yoga program on a yoga mat on the floor when they practiced it with a yoga instructor and on their bed when they practiced it at home.

The program consists of four parts: (1) adjusting external and internal conditions, (2) performing isometric yoga poses, (3) practicing deep relaxation and awakening, and (4) generalizing yoga-induced awareness to daily life. First, utmost attention was paid to external stimuli, such as temperature, humidity, sound, smell, and especially light, so that patients in a recumbent position could practice isometric yoga with minimal stress. When the patient practiced recumbent isometric yoga at the hospital, the room was equipped with brightness-adjustable ceiling lights that were adjusted by the instructor according to patient preference before starting the session. The instructor was mindful of the volume and tone of her voice. Patients were also asked to be mindful of these factors when they practiced at home to help promote an environment in which deep relaxation could be facilitated. Then, the patient was asked to loosen the tension of the whole body, especially the back, by adjusting the position of the pelvis and to focus on natural breathing and to put negative thoughts aside. Second, five isometric poses were practiced as described previously [10]. These poses were to be performed very slowly while breathing, with or without coordinated sounds, and with the patient using 30%–50% of their maximal muscular strength. We intentionally avoided poses that required strong stretches so that patients with FM could practice with minimal pain. Third, patients were asked to rest in a fully relaxed, recumbent position for several minutes and subsequently to awaken. The session was designed to take about 25 min. However, the program was modified on a patient-to-patient basis. For example, some patients, especially severe ones, decreased the number of repetitions per pose or skipped some poses depending on their physical condition.

#### Generalizing yoga-induced awareness to daily life

Fourth, in addition to regular practice of recumbent isometric yoga in the hospital and at home, on the day of the hospital visit, the patient’s therapeutic awareness evoked by yoga practice was shared with the physician, and patients were encouraged to generalize it to their daily lives. That is, the patients were encouraged to live their daily lives with the calm, relaxed, and peaceful feelings brought about by yoga and to develop the habit of listening to the “voice of the body” to become aware of the threshold that worsens their condition and triggers PEM and to be able to live peacefully within the limits of their energy level.

#### Monitoring of yoga practice

Patients were monitored in two ways: by a yoga instructor and through a “yoga diary”. When the patients practiced yoga at the hospital, their yoga instructor monitored and analyzed their practice and made changes if necessary. The patients were also asked to keep a “yoga diary”, if they could, in which they recorded the amount of time they practiced and any questions they had during the practice. On the day of the visit, the yoga instructor and the patient’s doctors reviewed the diary. According to the diaries, the patients in the yoga group practiced recumbent isometric yoga at home for about 4 times/week during the intervention period.

### Assessment of outcomes

#### Effects of recumbent isometric yoga on the level of functioning

To assess the long-term effect of recumbent isometric yoga on the level of functioning, the ME/CFS PS score of the Japanese clinical case definition was evaluated in both groups before and after the intervention period [17]. A decrease in PS score indicates an improvement in the functioning level.

#### Effects of recumbent isometric yoga on fatigue

Long-term effect: To assess the long-term effect of recumbent isometric yoga on fatigue, the Japanese version of the 11-item Chalder FS; a well-validated, self-reported Likert scale that measures the severity of fatigue in patients with ME/CFS, with a maximum score of 33; was applied to all patients [22, 23].

Short-term effect: To assess the effect of a single session of recumbent isometric yoga on fatigue, the fatigue (F) and vigor (V) scale scores of the Japanese edition of the Profile of Mood States (POMS) (Kaneko Shobo Co., Tokyo, Japan) [24, 25], a self-rated questionnaire, were compared immediately before and after the final session of recumbent isometric yoga with the instructor.

#### Subgroup analysis

To further assess if recumbent isometric yoga is effective in severe patients or patients with FM, changes in PS and the Chalder FS scores of severe patients (14 patients in the yoga group and 13 patients in the control group) and those with FM (6 patients in the yoga group and 7 patients in the control group) were compared in both groups. Furthermore, changes in the POMS F and V scores of the 14 severe patients and 6 patients with FM were compared immediately before and after the final session of recumbent isometric yoga.

#### Adverse events and benefits

Adverse events and benefits were monitored in two ways. First, at each hospital visit, the supervising doctor recorded any changes reported by the patient after practicing yoga with the instructor. Second, patients were asked to keep a yoga diary, if they could, in which they described how they felt after practicing. On the day of the hospital visit, the doctor reviewed the diary. After the intervention period, the patient recorded the physical and psychological changes and awareness induced by yoga and how they incorporated this awareness into their daily lives in the yoga diary.

### Sample size estimation

No previous studies have investigated changes in PS scores after an intervention. Therefore, to determine the sample size for this study, we referred to a previous study that assessed the changes in 14-item Chalder FS score after an 8-week seated isometric yoga intervention [9]. The Chalder FS scores in the yoga group decreased significantly, from 25.9 ± 6.1 to 19.2 ± 7.5. In another study, scores below 18 points, which is one standard deviation (SD) above the average for healthy individuals, were treated as remission. Based on these studies and considering a significant change due to intervention as a shift from 24 ± 5 to 18 ± 5, with a significance level of 0.05 and a power of 0.8, the required sample size was 15. In this study, we set the number of participants to 25, anticipating that the SD may be larger due to the inclusion of more severe patients from the previous study, as well as potential dropouts during the intervention period.

### Statistical analyses

Data are presented as mean ± SD. Normality in the data distribution was evaluated with the Kolmogorov–Smirnov test. Changes in PS and FS (△PS and △FS) are expressed as the differences in the corresponding values before and after the treatments. Differences in the variables were tested with the Mann–Whitney *U* test and an independent samples *t*-test, respectively. Changes in F and V were evaluated with a paired *t*-test. Differences in the categorical variables before and after the treatments were assessed with Fisher’s exact test or McNemar’s test, as appropriate. Effect sizes (ES) for significant differences were calculated with Cohen’s *d*, rank-biserial correlation, Cramer’s* V*, or Kendall’s *tau-b*, as appropriate. Statistical significance was set at *P* < 0.05, and all tests were two-tailed. Data were analyzed with SPSS v.21.0 and Jamovi v 2.2.5.

This study was conducted at Kyushu University Hospital and the International University of Health and Welfare Hospital, approved by the Institutional Review Boards of both hospitals (Approval number 87 from Kyushu University Hospital and 13-B-249 from the International University of Health and Welfare Hospital), and registered with the UMIN-CTR (UMIN 000023472 and 000030051). Written informed consent was obtained from all participants before enrollment. The study was conducted between August 4, 2016 and April 30, 2019.

## Results

### Participants

Among 358 eligible patients, 50 were initially enrolled for study. They were randomized in a 1:1 ratio into the yoga group or the control group. In the yoga group, one patient dropped out because of pregnancy just before the intervention started and one patient in the control group withdrew because of moving, leaving 24 participants in each group who completed the study (Fig. 1). In the yoga group, 20 were female with a mean (± SD) age of 37.9 ± 10.4 years (range 20 to 61 years). In the control group, 21 were female with a mean age of 39.4 ± 11.9 years (range 20 to 70 years). The mean (± SD) Chalder FS score of the yoga group was 23.1 ± 5.6, whereas that of the control group was 23.5 ± 5.4. The PS of the yoga group was between 4 and 8 (14 severe cases and 10 mild to moderate cases). By comparison, the PS of the control group was between 3 and 8 (13 severe cases and 11 mild to moderate cases). No patient was classified as PS 9 in either group. In the yoga group, 6 patients were diagnosed with FM as were 7 patients in the control group. These variables did not differ between groups (Table 2).

**Table 2:** Patient demographic characteristics

	Yoga group	Control group	*P*
Sex (male:female)	4:20	3:21	1.0*
Age, mean ± SD, years	37.9 ± 10.4	39.4 ± 11.9	0.644^#^
Chalder FS score, mean ± SD	23.1 ± 5.6	23.5 ± 5.4	0.814^#^
PS, n			
8	8	8	
7	6	5	
6	3	5	
5	3	3	
4	4	2	
3	0	1	0.908*
Severe (7–8), n (%)	14 (58%)	13 (54%)	
Mild to moderate (3–6), n (%)	10 (42%)	11 (46%)	0.771^x^
Comorbid FM, n (%)			
With FM	6 (25%)	7 (29%)	
Without FM	18 (75%)	17 (71%)	0.745^x^

The number (n) represents the number of patients of each PS. The data shown are the mean ± standard deviation

In this study, PS 7 and 8 were classified as severe and PS 3, 4, 5, and 6 were classified as mild to moderate

*FM* Fibromyalgia, *FS* Fatigue Scale, *n.s*. not significant, *PS* Performance status, *SD* Standard deviation

^*^Fisher’s exact probability test, ^#^independent-sample *t* test, ^x^*χ*^*2*^ test

#### Effects of recumbent isometric yoga on the level of functioning

To assess the long-term effects of recumbent isometric yoga on the level of functioning, we compared the PS of the yoga and the control groups before and after the intervention (Table 3). By Fisher’s exact test, the PS score was significantly different after the intervention (*P* < 0.001). The PS score of 6 patients in the yoga group decreased, whereas it was almost unchanged in the control group. To assess the changes in PS score, we compared the mean ∆PS scores. The Mann–Whitney U test found that the decrease in PS score in the yoga group was greater than that in the control group (*P* < 0.01), indicating that yoga significantly improved PS.

**Table 3:** Changes in PS and Chalder FS scores before (Pre) and after (Post) the intervention period in the yoga group and the control group

PS	Yoga group		Control group		P	ES
	Pre (n)	Post (n)	Pre (n)	Post (n)		
8	8	5	8	7	< 0.001*	0.76
7	6	4	5	5		
6	3	2	5	4		
5	3	4	3	3		
4	4	4	2	4		
3	0	3	1	1		
2	0	1	0	0		
1	0	1	0	0		
Severe (7–8)	14	9	13	12	0.031^#^	0.78
Mild to moderate (3–6)	10	15	11	12		
∆PS	−1.1 ± 1.3		−0.3 ± 0.5		0.005^×^	0.41
∆FS	−5.1 ± 4.8		−1.8 ± 3.4		0.009^&^	0.79

The number (n) represents the number of patients of each PS. The data shown are the mean ± standard deviation

*ES, *effect sizes were calculated using the *Kendall’s tau-b* and *Cramer’s V*, Rank biserial correlation, and Cohen’s D, respectively

*FS* Fatigue scale, *PS* Performance status

P, *p* value

*Fisher’s exact probability test

^#^paired samples McNemar’s test

^×^Mann–Whitney U test

^&^unpaired *t*—test

#### Effects recumbent isometric yoga on fatigue

##### Chalder FS

Change in the Chalder FS scores of the yoga and control groups were after the intervention to assess the long-term effects of recumbent isometric yoga on fatigue (Table 3). The Chalder FS score of both groups were significantly decreased after the intervention period, i.e. from 23.1 ± 5.6 to 18.0 ± 5.9 in the yoga group (*P* < 0.001, paired t-test) and from 23.5 ± 5.4 to 21.7 ± 6.3 in the control group (*P* < 0.05, paired t-test). However, an independent samples t-test found that the decrease in Chalder FS in the yoga group was greater than that of the control group (*P* < 0.01, unpaired t-test), indicating that yoga significantly improved the Chalder FS score.

##### POMS

We also assessed the short-term effects of recumbent isometric yoga on fatigue and vigor by comparing the POMS F and V scores before and after the final session of recumbent isometric yoga with the instructor (Table 4). Practicing recumbent isometric yoga significantly decreased the F score (from 22.6 ± 8.3 to 12.5 ± 6.7, *P* < 0.001, paired-t test) and increased the V score (from 17.6 ± 6.1 to 25.5 ± 8.7, *P* < 0.001, paired-t test).

**Table 4:** Changes in POMS F (fatigue) and V (vigor) scores obtained before (Pre) and after (Post) the intervention for participants in the yoga group

	Pre	Post	P	ES
F	22.6 ± 8.3	12.5 ± 6.7	< 0.001*	1.28
V	17.6 ± 6.1	25.5 ± 8.7	< 0.001*	−1.01

The data shown are the mean ± standard deviation

*F* Fatigue, *V* Vigor, *ES* effect size was calculated using *Cohen’s D*

P *p* value

*paired *t* test

#### Subgroup analysis

We conducted subgroup analyses to assess if recumbent isometric yoga was effective for severe patients and those with comorbid FM.

##### Severe patients

To assess the long-term effects of recumbent isometric yoga on the level of functioning and fatigue of the severe patients, we compared the changes in the PS and Chalder FS scores between the yoga and control groups after the intervention (Table 5a). The Mann–Whitney U test showed a greater tendency towards a decrease in PS score in the yoga group than was seen in the control group (*P* = 0.097). The Chalder FS score decreased significantly after the intervention period, from 25.8 ± 4.8 to 19.1 ± 5.9 (*P* < 0.001, paired t-test), in the yoga group, whereas it did not show a significant change, from 26.4 ± 4.6 to 25.2 ± 5.1, in the control group. An independent samples t-test demonstrated that the decrease in the Chalder FS of the yoga group was greater than that of the control group (*P* < 0.001). To assess the short-term effects of recumbent isometric yoga on fatigue and vigor, we compared the changes in the POMS F and V scores before and after the final session of recumbent isometric yoga with the instructor (Table 6). In severe patients, the F score was reduced significantly, from 25.4 ± 7.4 to 14.9 ± 7.7 (*P* < 0.001, paired-t test), and the V score was increased significantly, from 15.9 ± 5.4 to 25.5 ± 9.5 (*P* < 0.001, paired-t test).

**Table 5:** Differences in PS between the yoga group and the control group before (Pre) and after (Post) the intervention period in (a) severe patients and (b) those with comorbid FM (b)

PS	Yoga group		Control group		P	ES
	Pre (n)	Post (n)	Pre (n)	Post (n)		
(a) Severe patients						
8	8	5	8	7		
7	6	4	5	5		
6	0	2	0	1		
5	0	0	0	0		
4	0	3	0	0		
3	0	0	0	0		
Severe (7–8)	14	9	13	12		
Mild to moderate (3–6)	0	5	0	1		
∆PS	−1.0 ± 1.5		−0.1 ± 0.4		0.097*	0.31
∆FS	−6.6 ± 3.9		−1.1 ± 3.4		< 0.001^#^	1.48
(b) Patients with comorbid FM						
8	5	4	6	5		
7	1	2	0	1		
6	0	0	1	1		
5	0	0	0	0		
4	0	0	0	0		
3	0	0	0	0		
Severe (7–8)	6	6	6	6		
Mild to moderate (3–6)	0	0	1	1		
∆PS	−0.2 ± 0.4		−0.1 ± 0.4		1.0*	0.02
∆FS	−6.3 ± 3.6		0.3 ± 3.7		0.008^#^	1.81

The number (*n*) represents the number of patients of each PS. The data shown are the mean ± standard deviation

*ES* effect sizes were calculated using the Rank biserial correlation and Cohen’s *D*, respectively

*FS* fatigue scale, *PS* performance status, *n.s.* not significant

*P*, *p* value, *Mann-Whitney, *U* test, ^#^unpaired, *t*- test

**Table 6:** Differences in POMS F (fatigue) and V (vigor) scores in (a) severe patients and (b) those with comorbid fibromyalgia between the yoga group and the control group before (Pre) and after (Post) the intervention period

	Pre	Post	P	ES
(a) Severe patients				
F	25.4 ± 7.4	14.9 ± 7.7	< 0.001*	1.38
V	15.9 ± 5.4	25.5 ± 9.5	< 0.001*	−1.15
(b) Patients with FM				
F	26.2 ± 8.5	15.0 ± 7.4	0.018*	1.41
V	16.3 ± 5.9	24.0 ± 10.1	0.028*	−1.25

The data shown are the mean ± standard deviation (SD)

*F* Fatigue, *FM* Fibromyalgia, *V* Vigor, *ES* Effect size was calculated using *Cohen’s D*

P, *p* value

*paired *t* test

##### **Patients with comorbid FM**

We also compared the changes in the PS and Chalder FS scores of the yoga and control groups in patients with comorbid FM after the intervention (Table 5b). The Mann–Whitney U test indicated that there was no significant between group difference in the change in PS (*P* = 1.00). The Chalder FS score decreased significantly after the intervention period, i.e. from 30.2 ± 2.3 to 23.8 ± 4.8 (*P* < 0.01, paired t-test) in the yoga group, whereas it did not show a significant change, i.e. from 28.3 ± 4.8 to 28.6 ± 4.2, in the control group. An independent samples t-test suggested that the decrease in the Chalder FS score observed in the yoga group was greater than that of the control group (*P* < 0.01). As for the short-term effect of isometric yoga on fatigue and vigor, the F score was significantly reduced, from 26.2 ± 8.5 to 15.0 ± 7.4 (*P* = 0.018, paired-t test), and the V score was significantly increased, from 16.3 ± 5.9 to 24.0 ± 10.1 (*P* = 0.028, paired-t test) (Table 6b).

## Adverse events and benefits:

### Adverse events

No serious adverse events were reported after the patients became accustomed to the yoga procedures, including PEM. In the initial stage, one patient reported a transient deterioration of his condition, as described below. Furthermore, one patient declined to practice yoga with a yoga instructor once when she visited the hospital, not because she was tired, but because she felt that if she relaxed she would not be able to get up and go home.

### Benefits

After the intervention, we collected a “yoga diary” from 22 patients: 21 reported benefits of the recumbent isometric yoga program and described it as being useful and helpful (Table 7). These included both short-term physiological and psychological benefits, like improvements in fatigue, pain, insomnia, brain fog and “idling”, a state in which one is busy thinking about something and finding it difficult to stop, causing cognitive/emotional fatigue. Patients also reported long-term benefits, including that regular practice of recumbent isometric yoga enhanced bodily awareness and promoted behavioral changes to prevent PEM. Furthermore, some patients commented, “I am now able to take a break before I get tired,” “I have become more aware of my limitations and the thresholds that trigger crashes (i.e. PEM),” and “I have become more confident living within my energy level,” all of which suggested an improvement of self-efficacy to cope with this illness. In contrast, at the beginning of the intervention, one male patient with ME/CFS and FM (PS8) stated, “I usually feel better after practicing yoga, but when I practiced while feeling unwell, my condition worsened”, suggesting an inconsistent effect in the beginning.

**Table 7:** Feedback from patients who practiced recumbent isometric yoga

**(1) Improvement of symptoms:**
I became less tired. My body feels warm and relaxed. It became easier to breathe. My body stiffness has eased. I feel more energetic. My body pain eased. I used to have trouble falling asleep, but when I practice before going to bed, I can fall asleep easily
**(2) Improvement of psychological distress and brain fog:**
My mind is clearer. Brain fog has been alleviated. Idling (a state in which I am busy thinking about something and find it difficult to stop, even though I know my brain is tired) has been alleviated. By practicing yoga every day, I have been able to maintain a calm state after doing it. The uneasiness in my chest has calmed down. I feel warm and satisfied. My mind was always busy, but when I practice yoga, my mind becomes empty. It feels like my body and mind are melting
**(3) Release of repressed negative emotions:**
I cried a lot during yoga practice. Tears came out of my eyes even though I wasn't anxious, and afterwards I felt refreshed and energized
**(4) Enhanced awareness and behavioral changes that prevent PEM and promote recovery, leading to improvement of self-efficacy:**
I can now recognize when I am tense. Since I started practicing yoga, I have learned how to release tension. I used to be very impatient, but now I can relax. I can now listen to my body. I am now able to take a break before I get tired. I have become more aware of my limitations and the thresholds that trigger crashes. I have become more confident living within my energy level

## Discussion

The present study demonstrated that patients with ME/CFS who practiced recumbent isometric yoga together with conventional therapy reduced their PS scores more than those with conventional therapy alone. It also indicated that recumbent isometric yoga significantly improved the fatigue of patients with severe ME/CFS patients and those with comorbid FM. This fatigue-relieving effect was observed even after a single practice session when the patient became familiar with this procedure. To our knowledge, this is the first study to show a beneficial effect of yoga in severe ME/CFS patients and ME/CFS patients who also suffer from FM.

In our previous RCT, we demonstrated that the Chalder FS score was reduced more in a seated isometric yoga group than in controls [9]. However, in that study, the disease severity of the participants was moderate (PS 3–6), and two patients who needed assistance in their daily lives and who had difficulty remaining in a seated position for 30 min (PS 8) were excluded. Therefore, as the next step, we developed another yoga program that these patients could practice [10]. One who could not do seated yoga participated in this trial, and found it helpful, suggesting that the recumbent isometric yoga program would be beneficial for such severe patients who cannot practice the seated isometric yoga program.

The beneficial effects of recumbent isometric yoga for severe patients and those with FM may result, at least in part, from the characteristics of this program. First, patients can easily adjust resistance depending on their condition. This characteristic enabled the patients to practice this program on “bad days,” i.e., days when they felt unwell. Because fatigue severity varies from day to day, this characteristic is important for severe patients. Second, this program allows patients to practice in their bed. Because severe patients spend almost all their time in their beds, exercise that they can practice in bed provides a useful self-help strategy tool that improves their self-efficacy. Additionally, if patients feel fatigued after practicing a particular pose, they can take a pre-emptive rest in a recumbent position before the next pose. Furthermore, after practicing recumbent isometric yoga, they can rest with deeper relaxation, without moving. Third, the poses do not require strong stretches or physical flexibility. We intentionally chose such poses to prevent overstretching that may cause worsening of the pain of patients with FM. Fourth, this program consists of isometric muscular contractions. The isometric exercise technique, which starts at a naturally stationary position with minimal resistance, is known to induce post-isometric relaxation, improve the tenderness of tense muscles, and produce immediate pain relief in patients with myofascial pain [26, 27]. The first and second characteristics are useful for preventing exacerbation of fatigue during practice and for avoiding PEM. The third and fourth characteristics are useful for patients with FM.

From this study, we cannot identify the underlying mechanisms mediating the beneficial effects, such as improved functional levels, reduced fatigue, and increased vigor. Our previous study demonstrated that a single session of seated isometric yoga practice reduced cortisol and tumor necrosis factor-α and increased dehydroepiandrosterone sulfate in the blood [28]. It also increased the high frequency power of heart rate variability [28]. These findings suggest that even a single session of seated isometric yoga provides anti-stress and anti-inflammatory effects and increases parasympathetic nervous system activity. However, after two months of practicing seated isometric yoga, baseline values of these parameters did not differ between pre- and post-intervention. Instead, the questionnaires to evaluate depression and alexithymia scores decreased two months after practice [29], suggesting that psychological improvement may play a more important role as a chronic effect of seated isometric yoga. Therefore, the beneficial effects of recumbent isometric yoga may be due to the same factors as those observed in patients who practiced seated isometric yoga.

Furthermore, we found that regular practice of recumbent isometric yoga increased several circulating microRNAs [30] and increased the volume of the prefrontal cortex [31] in patients with ME/CFS. It also improved postural orthostatic tachycardia syndrome (POTS) in these patients [32]. It is well known that deconditioning is one of the causes of POTS [33–35]. Because some patients were bedridden for most of the day, deconditioning may have contributed to POTS in these patients. Recent studies have demonstrated that physical exercise is useful in the treatment of POTS [36, 37]. However, it is difficult to introduce exercise therapy to ME/CFS patients because they rapidly become exhausted and develop PEM after minimal physical exertion [38, 39]. Because patients can easily adjust muscular resistance according to their condition during yoga practice, regular practice of recumbent isometric yoga may help counteract deconditioning in ME/CFS patients and lead to improvement of POTS without causing PEM. All of these changes may also contribute to the beneficial effects of recumbent isometric yoga.

 Additionally, we hypothesized that the practice of an isometric yoga program would provide psychological and behavioral benefits, such as increasing interoceptive awareness and cultivating a “therapist self” for better coping [9, 10, 30], because these features have been demonstrated to contribute to improvements in physical symptoms such as pain, functionality and quality of life in patients with fibromyalgia, a common comorbidity of ME/CFS [40, 41]. In fact, feedback from the patients indicates that practicing recumbent isometric yoga not only improved physical and psychological symptoms but also helped them to recognize the limits and thresholds that trigger PEM and to learn appropriate coping skills. These changes are beneficial for patients with ME/CFS because it is important for them to avoid PEM and to live within their energy limit, i.e. to learn to pace themselves [38, 39, 42]. Such beneficial changes may be related to how yoga was instructed because patients are asked during yoga practice to pay mindful attention to their inner bodily sensations (interoception and proprioception), such as breathing and muscle tension/relaxation [43]. Additionally, after practicing, patients are also encouraged to generalize the calm, peaceful and relaxed state of mind and awareness brought about by yoga into their daily lives. These may help patients learn and develop habits for optimal coping skills, including pacing.

**Fig. 2:**
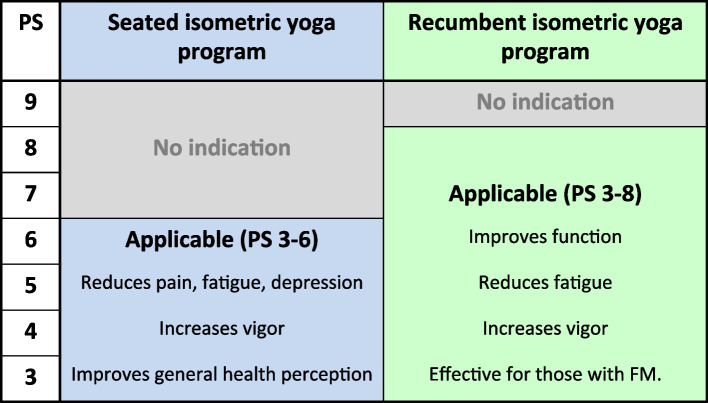
Indications of seated and recumbent isometric yoga programs for patients with ME/CFS with different PS scores, created based on data from previous studies [9, 10, 29] and the present study. For those with a PS score of 9, neither seated nor recumbent isometric yoga is recommended. For those with PS scores of 7 and 8, a recumbent, but not a seated, isometric yoga program is recommended. However, for those with a PS score of 8, it is advised to receive personal guidance initially from a physician or a yoga instructor. For those with PS scores of 3, 4, 5, and 6, both seated and recumbent isometric yoga programs are applicable. However, the authors recommend a recumbent isometric yoga program, based on our previous studies. In any case, isometric yoga is considered an option for additional therapy after medical, behavioral, and psychological treatments have been initiated by specialists.

Based on the present findings and those of previous studies, an indication for seated and recumbent isometric yoga for patients with ME/CFS that considers their PS is illustrated in Fig. 2.

## Limitations

This study has several limitations. First, although patients felt the usefulness and benefit of recumbent isometric yoga, the PS scores of some patients did not change. It will be important to observe the effects of the recumbent isometric yoga program for a longer period of time, but this finding indicates that the recumbent isometric yoga program alone has limitations to its effectiveness. Second, in this study, no extremely severe patients (PS 9) were included. Therefore, although recumbent isometric yoga was effective for severe patients (PS 7 or 8), the efficacy for extremely severe patients who need more help in their daily lives will require further study to determine if it is also effective for such patients. Third, the mechanisms of the beneficial effects are speculative: we are now analyzing changes in blood biomarkers and autonomic nervous system indices related to the practice of recumbent isometric yoga. Fourth, the long-term effects of isometric yoga (for more than 12 weeks) are not known. Considering the nature of this illness, it is not uncommon for patients to struggle with it for years. So far, however, only one case report has described the results of a two-year follow-up of patients with ME/CFS following a yoga-based lifestyle intervention [8]. Therefore, it is important to assess the longer-term effects of isometric yoga, e.g., for more than a year, in future studies.

Despite these limitations, this study is noteworthy in that a therapeutic option is proposed for the first time for patients with ME/CFS who are for the most part bedridden and for those with FM.

## Conclusions

The results of this study showed that recumbent isometric yoga decreased the fatigue of these patients with ME/CFS, including severe patients and patients with FM. Recumbent isometric yoga may be beneficial as a self-help strategy or adjunct therapy for severe ME/CFS patients and patients with ME/CFS and FM. Further studies are needed to determine the mechanisms by which isometric yoga improves the fatigue of these patients.

## Data Availability

Not applicable.

## Abbreviations

ANOVA: Analysis of variance
ES: Effect size
F: Fatigue
FM: Fibromyalgia
FS: Fatigue scale
ME/CFS: Myalgic encephalomyelitis/chronic fatigue syndrome
PEM: Post-exertional malaise
POMS: Profile of Mood States
PS: Performance status
RCT: Randomized, controlled trial
SD: Standard deviation
V: Vigor
